# Cures by Lycopodium

**Published:** 1892-08

**Authors:** 

**Affiliations:** Hamburg, Germany


### CURES BY LYCOPODIUM.

DR. HESSE, HAMBURG, GERMANY.

Translated by A. McNeil, M. D., San Francisco.

### I.

An emaciated woman of sixty has suffered for two years from vomiting of food that occurs after every meal. She had bitter taste. She is always *full of flatus* and has constipation. She *must lie on her back with her head high.*

She received on September 17th, 1889, five powders of Lycopodium$^{30}$, one to be taken each evening.

October 5th, vomiting has ceased and her appetite is better. Placebo.

I did not hear from my patient till May 3d, 1890, when I was informed that she had remained perfectly well.

### II.

Frau M., *ætat.* forty, has complained for years of *swelling of the abdomen toward evening*, often from about 3 P. M. *Frequent urination at night.* Pain in the region of right ovary since a confinement six years ago. *Much flatus; cabbage, beans, and peas disagree; she can lie only on her back; swelling of the feet toward evening; cold feet.*

October 11th, 1889, Lycopodium$^{30}$, five powders, one every evening.

November 11th, is considerably improved in every respect. She would not have returned on account of the abdominal distention, swollen feet, etc., if she had not been for some time temporarily troubled with other complaints. She suffers much from coryza, toothache, and slight pains in swallowing when she catches cold. She has suffered much from nose-bleed also.

I again gave her Lycopodium$^{30}$, six powders, one each evening. I have in my notes that Sulphur is the most appropriate remedy to remove the pains which resist Lycopodium. However, she did not return, but I learned incidentally on April 1st that she has had nothing of which to complain since my last treatment.

### III.

Frau Gr., *ætat.* forty-eight, has suffered for years from gastric troubles. *Everything she ate turned to gas, particularly fruit, black bread, beans, and peas. Stomach and abdomen swell toward evening.* Appetite moderate, no thirst. *Can lie only on her back.* Tearing pains in the soles of the feet. Heat in the feet, they are never cold. Neck becomes stiff on catching cold, particularly if the weather is rainy. Menses every three weeks, often black and lumpy.

November 12th, 1890, Lycopodium$^{30}$, a powder every week for six weeks.

January 2d, 1892, I find in my notes that she did not return, as the first powder helped her.

Kunkel characterizes Lycopodium briefly and clearly as follows :

Dry, withered skin, tendency to eruptions, very conspicuous is its effect in flatulent complaints which appear in the afternoon (characteristic from four to eight), with congestion to the head and heat of the face with cold or wet cold feet. The air of a warm room is unpleasant. Sleeps on his back with his head very high. To lie with his head low or on his side is impossible, and warm covering of the head also, and covering of the head is often intolerable, and also sitting long or eating enough disposes to formation of acids in stomach and sour vomiting.

Lycopodium is one of the most sharply characteristic remedies, and in cases where indicated, the beneficial effect may be predicted and the action of the thirtieth potency demonstrated to the unbelieving. A sensitive lady, whom I cured with Lycopodium (except a few doses of Sepia in the latter part of the treatment) of an asthma of years' standing, after every powder of the thirtieth had been attacked by an immense distention of the abdomen which lasted several days. It was a form of asthma such as Farrington, whom one never opens without learning something from, describes, under Nux-vomica. He says: "Nux many times is useful in asthma, but it is that which arises from gastric disturbance and is usually not a purely nervous disturbance. It is attended by a feeling of fullness and pressure in the stomach, which comes particularly after a good meal, and he must loosen all the clothing about the hypochondrium. The abdomen is distended by flatus. Eructations relieve their asthmatic condition. Carbo-veg. and Lycopodium may be indicated in asthma with abdominal irritation with pronounced flatulence." In the case of the above-mentioned lady my decision for Lycopodium was made on account of the aggravation in the afternoon and evening and amelioration, and which was the only amelioration, by walking in the open air.

At present I have a cigarmaker under my care whose symptoms correspond to the above description of Nux asthma. Cough and shortness of breath; worse after midnight, and by moving; better by eructations; stomach and abdomen always distended; feeling of a rope about the bowels that troubles him very much. I gave him without benefit and very incorrectly Kali-carb. on account of the aggravation about 3 A. M. On my following visit he told me that he took the medicine about 4 A. M., then he became extremely cheerful, and afterward slept again and awakened after it relaxed and tired. This, with the distention of the abdomen, decided me to give Nux-vomica. Some days afterward I read in *Farrington* the above points. This shows clearly how with advantage one leaves the name of the disease out of consideration and regards only the totality of the symptoms, and how the symptoms, kept in the background by the patient, may point out the proper remedy.

Often with Lycopodium we find only an aggravation toward evening, but often also exactly between 4 and 8 o'clock. A pulmonary hemorrhage that had continued eight days was immediately stopped by Lycop. Decisive for its selection was fever exactly from 4 to 8 P. M.—intolerance of a warm room. The latter is seldom or never absent when Lycop. is *the* remedy. The desire for fresh air is uncommonly characteristic and often so great that even in an unheated room the doors and windows must be kept open. Several years ago Lycopodium did me good service in many severe cases of diphtheria when the intolerance of a warm room and fever appearing or increasing from 4 to 8 P. M. decided me. Soon after I learned that with our American colleagues Lycopodium took a justly entitled place in diphtheria which had its seat in the right tonsil or started from it, with the nose stopped up. It is seldom in these cases that there are not other symptoms of Lycopodium.

It is well known that it was with the drug picture of Lycopodium that Dr. Chapman, in 1889, demonstrated that Homœopathy had a unifying principle, and that allopathy was destitute of any such.

[The particulars are so well known to all American homœopaths that it is a work of supererogation to repeat the experiment.—Trans.]

